# Reply to Mafaciolli and Pasqualotto

**DOI:** 10.1093/cid/ciaa104

**Published:** 2020-02-01

**Authors:** Paul E Verweij, Johan Maertens, Sharon C-A Chen, Peter G Pappas, J Peter Donnelly

**Affiliations:** 1 Center of Expertise in Mycology Radboudumc/CWZ, Nijmegen, The Netherlands; 2 Department of Hematology, University Hospitals Leuven, Leuven, Belgium; 3 Department of Microbiology, Immunology and Transplantation, K.U. Leuven, Leuven, Belgium; 4 Centre for Infectious Diseases and Microbiology, Laboratory Services, Institute of Clinical Pathology and Medical Research, Westmead Hospital, University of Sydney, Sydney, Australia; 5 Department of Medicine, Division of Infectious Diseases, University of Alabama at Birmingham, Birmingham, Alabama, USA; 6 Department of Hematology, Radboudumc, Nijmegen, The Netherlands

To the Editor—Mafaciolli and Pasqualotto [1] question the proposal in the recently published update of the Infectious Diseases Group of the European Organization for Research and Treatment of Cancer and the Mycoses Study Group Education and Research Consortium (EORTC/MSGERC) definitions of invasive fungal diseases to adopt a cutoff of 1.0 for the galactomannan index (GMI) for both serum and bronchoalveolar lavage (BAL) fluid specimens [2]. They are particularly concerned that this threshold differs from the 0.5 recommended by the manufacturer. In fact, the group involved in the 2008 revision actually failed to reach a consensus on the cutoff for GM and so decided to place the onus on the manufacturer and to adopt whatever threshold values they recommended [3]. Originally, the Platelia *Aspergillus* assay was released with a cutoff index of 1.5, but after review, it was decided to lower this to 0.5 to maximize sensitivity [4]. This increased the utility of galactomannan in clinical practice by allowing cases of invasive aspergillosis to be identified earlier. However, the consensus definitions of the EORTC/MSGERC are actually intended to facilitate clinical research in invasive fungal disease and to enable comparisons between studies. They are not, and never were, intended to direct or guide patient care [2].

By way of illustration, since the publication of the 2008 EORTC/MSG definitions update, two major clinical trials of antifungal agents have actually used other cutoff GMIs to enroll patients with invasive aspergillosis. In the study of Marr *et al*, mycologic criteria for a diagnosis of probable invasive aspergillosis were satisfied when a GMI of at least 0.5 was determined in 2 serum samples or in a single BAL fluid [5]. By contrast, in the study of Maertens *et al* the mycological criteria of probable invasive aspergillosis were met after serum GM test yielded a GMI ≥ 0.7 in a single sample or a GMI ≥ 0.5 in two consecutive sera. Detecting GM in BAL fluid was not accepted as a mycologic criterion because the assay had not yet been approved by the US FDA for this specimen [6].

The decision to adopt a GMI of ≥ 1.0 was not taken lightly but was chosen to help ensure enrollment in clinical trials of patients with a high-likelihood of having invasive aspergillosis. The higher GMI will increase the specificity of the test by lowering the rate of false-positivity although this will inevitably result in a lower sensitivity as was pointed out by Leeflang *et al* [7]. There are even sound arguments for returning the threshold to its original level of 1.5 or even higher to increase the positive predictive value further still. However, as clinical trials on invasive aspergillosis are difficult enough to conduct a very high cutoff could severely limit the number of patients that would be eligible for enrollment. Therefore a cutoff of 1.0 was chosen as the best compromise, both for serum and for BAL-fluid. We hope that the current recommended GMI cutoff will help to further standardize clinical studies in patients with invasive aspergillosis and allow greater inter-study consistency.

## Notes

***Potential conflicts of interest***. S. C. reports grants from Merck Sharp & Dohme (MSD) Australia, outside the submitted work. J. P. D. reports consulting fees from F2G Ltd and Gilead Sciences, and speaking fees from Gilead Sciences and Pfizer, outside the submitted work. P. V. reports grants from Gilead Sciences, MSD, Pfizer, and F2G, and nonfinancial support from OLM and IMMY, outside the submitted work. All other authors report no potential conflicts. All authors have submitted the ICMJE Form for Disclosure of Potential Conflicts of Interest. Conflicts that the editors consider relevant to the content of the manuscript have been disclosed.
